# Supplementary material for: ssDNA recombineering boosts in vivo evolution of nanobodies displayed on bacterial surfaces
Source: Commun Biol. 2021 Oct 7;4:1169. doi: 10.1038/s42003-021-02702-0 (PMC8497518; doi:10.1038/s42003-021-02702-0)
Supplement: Supplementary file 2 — Supplemental Information [file 42003_2021_2702_MOESM2_ESM.pdf]

SUPPLEMENTARY INFORMATION

ssDNA recombineering boosts *in vivo* evolution of nanobodies displayed on bacterial surfaces

by

Yamal Al-ramahi[1], Akos Nyerges[2ꭗ], Yago Margolles[3], Lidia Cerdán[3], Gyorgyi Ferenc[2], Csaba Pál[2], Luis Ángel Fernández[3] and Víctor de Lorenzo[1]

[1]*Systems and Synthetic Biology Department,* [3]*Department of Microbial Biotechnology, Centro Nacional de Biotecnología (CNB-CSIC), Campus de Cantoblanco, Madrid 28049, Spain;* [2]*Synthetic and Systems Biology Unit, Institute of Biochemistry, Biological Research Centre, Szeged H-6726, Hungary*

* Correspondence to: vdlorenzo@cnb.csic.es, lafdez@cnb.csic.es

Supplementary Table S1. Strains used in this work

Strain *Escherichia coli*	Description	Reference
DH5α	*supE44, ΔlacU169 (φ80 lacZΔM15), hsdR17, recA, endA1, gyrA96,* thi^{-1}, *relA1*	[1]
BL21(DE3)	*hsdS, gal* (λcI^{ts}*857, ind1, Sam7, nin5, lacUV5-T7* gene 1)	[2]
HB2151	*nalr thi-1 ara ∆(lac-proAB) [F' proAB+ lacIq lacZ(M15]*	[3]
WK6	F' *lacIq ∆(lacZ)M15 proA+B+ ∆(lacproAB) galE rpsL*	[4]
EcM1luxSATir	MG1655*∆fimA-H ∆flu::*P_{N25}*-SAtir ∆matB::*P_2*-luxCDABE*	[5]
EcM1luxSATir (pORTMAGE-3)	Bacterial strain that expresses constitutively the Nb[1] TD4 and enables its display in the cell surface. This strain was used as parental for the generation of libraries. The plasmid pORTMAGE-3 is to enhance the efficiency of recombineering events.	This work
BL21 DE3 (pET28a_TirM EPEC)	Used for the overproduction of the antigen EPEC TirM.	
BL21 DE3 (pET28a_TirM EHEC)	Used for the overproduction of the antigen EHEC TirM.	[4, 6]
EcM1H107Y	Strain isolated from the library. Bears the cassette in the genome for the expression of the Nb with the replacement H107Y.	This work
EcM1T108R	Strain isolated from the library. Bears the cassette in the genome for the expression of the Nb with the replacement T108R.	This work
EcM1D116G	Strain isolated from the library. Bears the cassette in the genome for the expression of the Nb with the replacement D116G.	This work
HB2151(pVdL9.3_Vamy)	Strain that bears the plasmid for the overexpression of the Nb Vamy, which we used as the unspecific control antibody.	[7]
HB2151(pVdL9.3_TD4)	Strain that bears the plasmid for the overexpression of the Nb TD4.	
HB2151(pEHLYA_H107Y + pVdL9.3)	Strain carrying the plasmids for the expression and secretion of the Nb H107Y.	This work
HB2151(pEHLYA_T108R + pVdL9.3)	Strain carrying the plasmids for the expression and secretion of the Nb T108R.	This work
HB2151(pEHLYA_D116G + pVdL9.3)	Strain carrying the plasmids for the expression and secretion of the Nb D116G.	This work
WK6 (pCANTAB6_VhhTD4)	Produces the plasmid pCANTAB6_VhhTD4 used afterwards as template to obtain, by site directed mutagenesis, the different Nb gene variants which were cloned in pVdL9.3 plasmid and expressed in HB2151.	[4]
WK6 (pCANTAB6_H107Y)	Used to obtain the plasmid pCANTAB6_H107Y that was digested SfiI/NotI to clone the gene of the Nb H107Y in pVdL9.3 plasmid.	This work
WK6 (pCANTAB6_T108R)	Used to obtain the plasmid pCANTAB6_T108R that was digested SfiI/NotI to clone the gene of the Nb T108R in pVdL9.3 plasmid.	This work
WK6 (pCANTAB6_D116G)	Used to obtain the plasmid pCANTAB6_D116G that was digested SfiI/NotI to clone the gene of the Nb D116G in pVdL9.3 plasmid.	This work

[1] Nb, nanobody

Supplementary Table S2. Plasmids used in this work

Name	Description	Reference
pORTMAGE3	Plasmid for the transient expression (controlled by temperature sensitive cI857 repressor and pL promoter) of λ Red Exo, Beta, Gam, and the MutL E32K protein, which confers a dominant mutator phenotype, to enhance the establishment of nucleotide replacements in the genome during DIvERGE. Confers resistance to kanamycin.	[8]
pET28a-TirMEHEC	For the overproduction of the antigen EHEC TirM and subsequent purification. Confers resistance to kanamycin.	[4]
pET28a-TirMEPEC	For the overproduction of the antigen EPEC TirM and subsequent purification. Confers resistance to kanamycin.	[4]
pCANTAB6_VHHTD4	Bears the gene for VHHTD4. This plasmid was used as template for the transplantation of the genes for new Nb variants by codon replacement. Bears a pUC-ori and confers resistance to ampicillin.	[4]
pEHLYA5_Vamy	Used for the purification of the Nb Vamy, specific for alpha amylase. Confers resistance to ampicillin.	[9]
pCANTAB6_NbH107Y	Used as source of the gene encoding the NbH107Y by digestion with SfiI and NotI. Confers resistance to ampicillin.	This work
pCANTAB6_NbT108R	Used as source of the gene encoding the NbT108R by digestion with SfiI and NotI. Confers resistance to ampicillin.	This work
pCANTAB6_NbD116G	Used as source of the gene encoding the NbD116G by digestion with SfiI and NotI. Confers resistance to ampicillin.	This work
pVdL9.3	Encodes HlyB and HlyD components of the haemolysin secretion system, for controlled expression under the Plac promoter. pSC101-ori. Confers resistance to chloramphenicol.	[7]
pVdL9.3_Vamy	Used for the purification of the Nb Vamy, specific for alpha amylase, from *E. coli* culture supernatants using the haemolysin secretion system. Confers resistance to chloramphenicol.	[7]
pVdL9.3_TD4	Used for the purification of the Nb TD4, specific for the antigen TirM EHEC, from *E. coli* culture supernatants using the haemolysin secretion system. Confers resistance to chloramphenicol.	[7]
pEHLYA_NbH107Y	Used in combination with the plasmid pVdL9.3 for the purification of the NbH107Y from *E. coli* culture supernatants using the haemolysin secretion system.	This work
pEHLYA_NbT108R	Used in combination with the plasmid pVdL9.3 for the purification of the NbT108R from *E. coli* culture supernatants using the haemolysin secretion system.	This work
pEHLYA_NbD116G	Used in combination with the plasmid pVdL9.3 for the purification of the NbD116G from *E. coli* culture supernatants using the haemolysin secretion system.	This work

Supplementary Table S3. Oligonucleotides used in this work

Oligo ID	Name	Sequence / description	Tm ºC
1	TD4_T108R_F	CATGGGACCGCGCCATATTGGCACAgGCCCATCCCTACTCTCTCCGAAG	87
2	TD4_T108R_R	CTTCGGAGAGAGTAGGGATGGGCcTGTGCCAATATGGCGCGGTCCCATG	87
3	H107Y_Fwd	GACCGCGCCATATTGGtACACGCCCATCCCTACT	79
4	H107Y_Rev	AGTAGGGATGGGCGTGTaCCAATATGGCGCGGTC	79
5	D116G_Fwd	CCCTACTCTCTCCGAAGgTAAGTATTTCTACTGGG	75
6	D116G_Rev	CCCAGTAGAAATACTTAcCTTCGGAGAGAGTAGGG	75
7	pCANTAB6_F	CCAGTACACTCCTGTATCATCAAAAGCC	68
8	pCANTAB6_R	CTCTTCGCTATTACGCCAGC	60
9	CDRI_Fwd	CTCAGGTGCAGCTGGTGGACG	67
10	CDRII_Midd_R	CAGGCTGTTCATCTGCAGATATATCGTG	68
11	CDRIII_Rev	CAGCTGCATCCTCTTCTGAGATGAG	67
12	CheckSAtir_F	CGCCTATGACCGTAATGGCAATAGCTCTAACAATGTACAGC	78
13	CheckSAtir_R	TCAGAAGGTCACATTCAGTGTGGCCTGACCGTTATACC	78
14	tirDSF	CTCGGTGCAGGCTGGA	56
15	tirDSR	CCGCTGAGGAGACGGTGACCTG	69
16*	tirRM1	GGGTCTCTAACACTCTCCTGTGTAGCCTCTGGAGCCGCCTACAGTACGAACTTGTT GGGCTGGTTCCGCCAGGCTCCAGGGAAGGAGCGC	-
17*	tirRM2	GGGGTCGCATCTATTTATCGTGGTAATAGTGCCACGAACTATGCCGACTCCGTGA AGGGCCGATTCACCATCTCCCAAGACAAGACCAAA	-
18*	tirRM3	CATGTACTACTGTGCACATGGGACCGCGCCATATTGGCACACGCCCATCCCTACT CTCTCCGAAGATAAGTATTTCTACTGGGGCCAGGG	-

*underlined sequence regions were synthesized with soft-randomized monomer mixture as follows: A = 98.5%A+0.5%C+0.5%T +0.5%G; C = 98.5%C+0.5%A+0.5%T +0.5%G; G = 98.5%G+0.5%C+0.5%T +0.5%A and T = 98.5%T+0.5%C+0.5%A +0.5%G.

Supplementary Table S4. Amino acid sequence of the different nanobodies

Nb TD4	MAQVQLVDAGGGSVQAGGSLTLSCVASGAAYSTNLLGWFRQAPGKEREGVASIYRGNSATNYA DSVKGRFTISQDKTKYTIYLQMNSLKPEDSAMYYCAHGTAPYWHTPIPTLSEDKYFYWGQGTQVT VSSAA
Nb H107Y	MAQVQLVDAGGGSVQAGGSLTLSCVASGAAYSTNLLGWFRQAPGKEREGVASIYRGNSATNYA DSVKGRFTISQDKTKYTIYLQMNSLKPEDSAMYYCAHGTAPYWYTPIPTLSEDKYFYWGQGTQVT VSSAA
Nb T108R	MAQVQLVDAGGGSVQAGGSLTLSCVASGAAYSTNLLGWFRQAPGKEREGVASIYRGNSATNYA DSVKGRFTISQDKTKYTIYLQMNSLKPEDSAMYYCAHGTAPYWHRPIPTLSEDKYFYWGQGTQV TVSSAA
NbD116G	MAQVQLVDAGGGSVQAGGSLTLSCVASGAAYSTNLLGWFRQAPGKEREGVASIYRGNSATNYA DSVKGRFTISQDKTKYTIYLQMNSLKPEDSAMYYCAHGTAPYWHTPIPTLSEGKYFYWGQGTQV TVSSAA

Supplementary Figure S1. Nucleotide changes through the anti-TirM^{EHEC} V_{HH} sequence following 10 DivERGE cycles of mutagenic recombineering exposed with PacBio data.

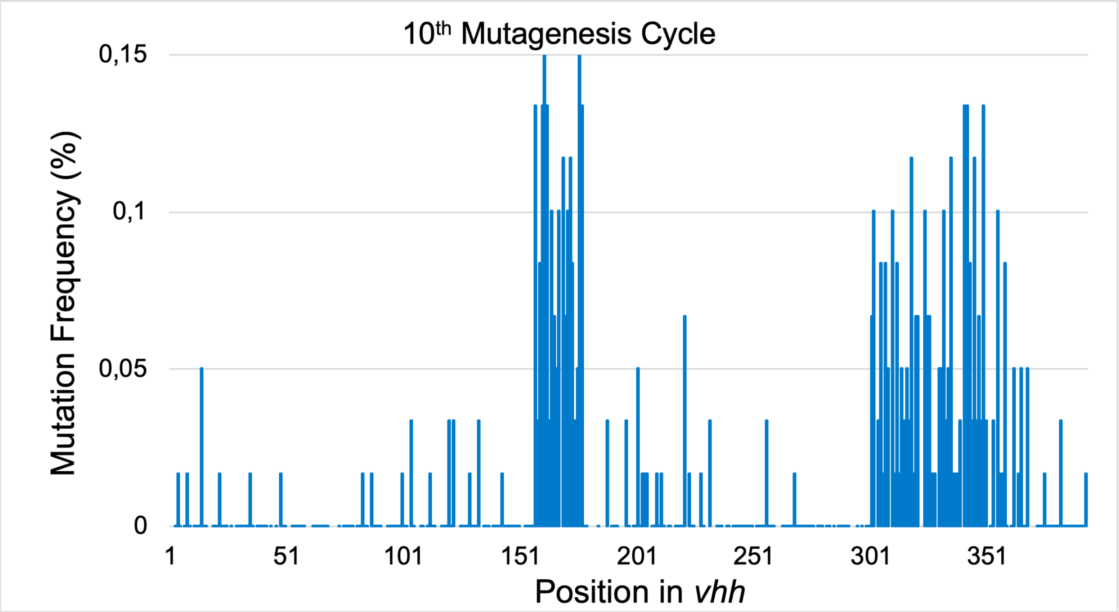

Following mutagenesis as indicated in the main text, DNA sequences of the V_{HH} genes were amplified from the merged genomic DNA and submitted to Pacific Biosciences Circular Consensus (PacBio CCS) sequencing. Note intensification of mutants in CDR2 (nt 157-177) and CDR3 (nt 301-360) as compared to changes in CDR1 (nt 85-111).

Supplementary Figure S2. Purification of TirM EPEC and EHEC antigens.

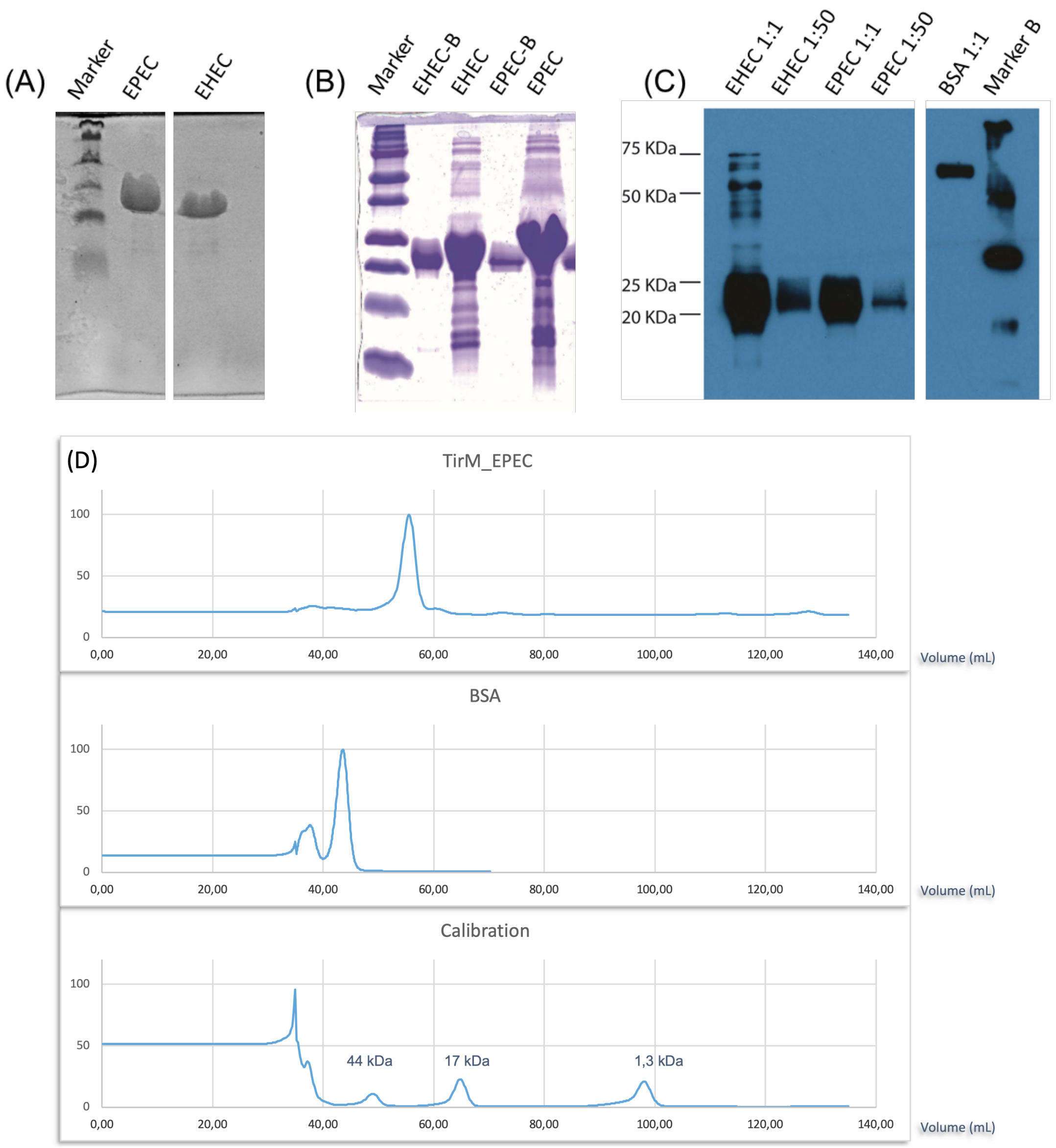

(A) Protein verification by SDS-PAGE. The antigens EPEC and EHEC were checked during the different preparation steps. (A) SDS-PAGE after purification by Immobilized Metal Affinity Chromatography (IMAC) and dialysis. Marker band sizes are 7.1, 20.6, 28.9, 34.8, 49.1, 80, 124, and 209 kDa. (B) SDS-PAGE with samples of the antigens biotinylated, EHEC-B and EPEC-B, and without biotinylation, EHEC and EPEC. (C) Western blot showing different sample dilutions of the biotinylated antigens. (D) Dimeric behaviour of purified TirM_EPEC. Gel-filtration chromatograms performed in a HiLoad 16/600 Superdex 75 column of affinity purified his-tagged TirM_EPEC protein (top), BSA (middle), and molecular weight (MW) markers labeled in kDa (bottom). BSA has a MW of ~66 kDa and a similar size in gel filtration, corresponding to a monomer in solution. His-tagged TirM_EPEC has a MW of ~15 kDa and an apparent size of ~30 kDa in gel filtration, which corresponds to a dimer in solution.

Supplementary Figure S3. Effect of the MACS-enrichment process.

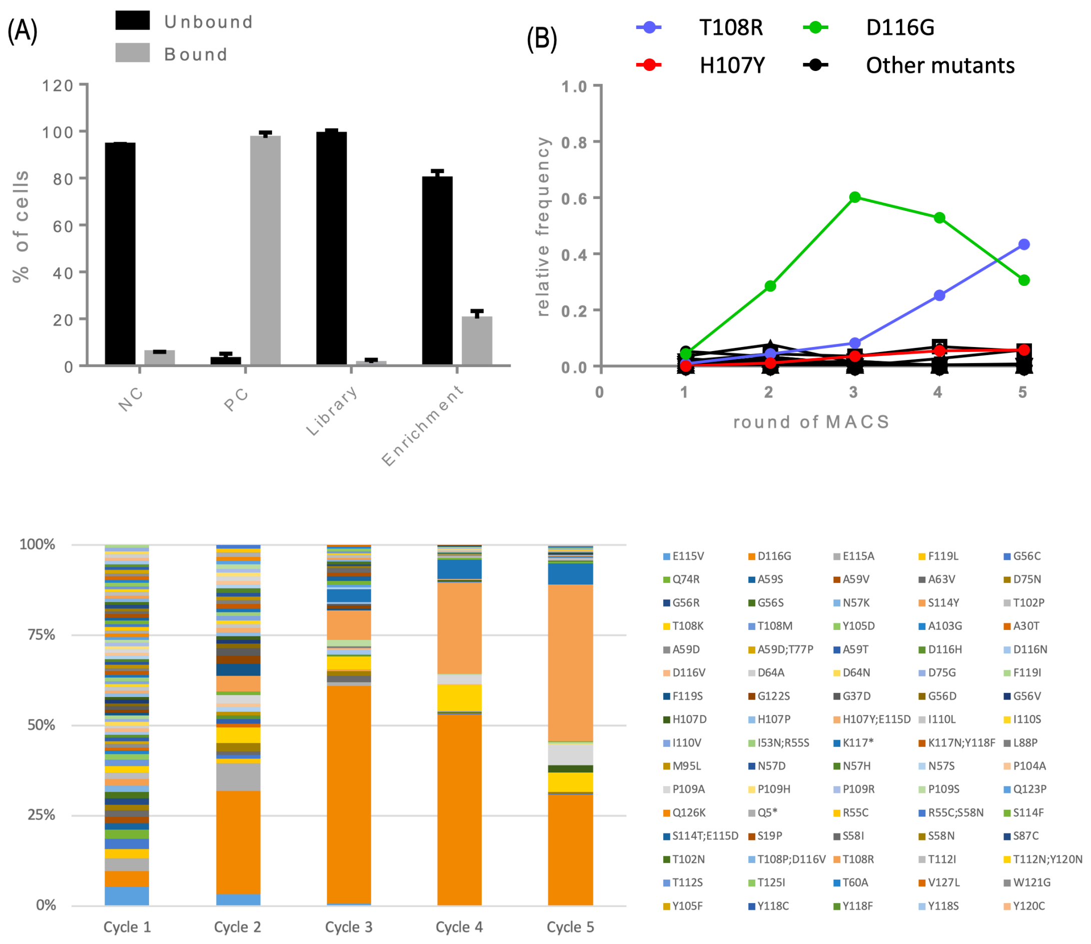

(A) NC corresponds to a strain that expresses and displays a nanobody that is specific for GFP recognition and it is used as unspecific control for this experiment. PC (positive control) corresponds to the parental nanobody TD4 and it is used as control of a positive interaction. Almost all of the cells that display the parental nanobody can bind specifically to the former antigen (PC), but nevertheless, the number of cells from the library that are able to bind the new antigen is extremely low (Library). In contrast, after the enrichment, the bias towards the ability to bind the new antigen becomes evident as shown by the increase in cells that bind to EPEC TirM after the enrichment (Enrichment). (B) Based on mutant counts found by sequencing with PacBio technology. The frequency of mutant clones after each round of library enrichment is represented (number of times a sequence appeared/number of times all the mutant sequences appeared). Compared to the rest of the mutants in the library, the abundance of clones producing any of the nanobody T108R and D116G is specially increased during the enrichment. The trend is represented in colors for the three clones that were isolated for deeper characterization in this work and in black for the rest of mutants. (C) Detail of the progress of V_{HH} population composition along cycles of immunomagnetic enrichment of $TirM^{EPEC}$-binding bacteria. The bar diagrams show the frequencies of the new Nb mutants displayed by bacteria captured in subsequent MACS cycles. The figure illustrates how among the diversity of mutants more evenly present in cycle 1, some become predominant above the other mutants after the immunomagnetic enrichment. In cycle 1, clones in the library are more evenly distributed compared with cycle 4 and cycle 5, were three mutants (H107Y, T108R, and D116G) comprise >80% of the library.

Supplementary Figure S4. Verification of the Nb variants by SDS-PAGE**.**

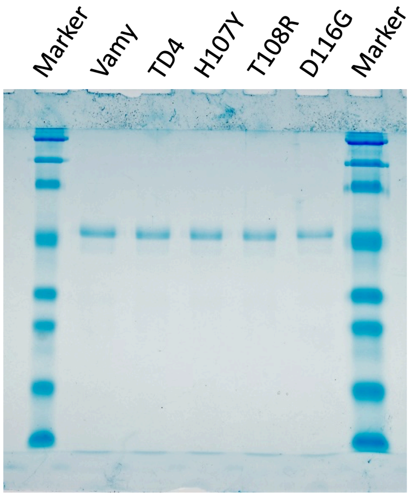

Nb variants after they were purified and concentrated were checked before they were used in ELISA. From each Nb sample 6 µg were loaded. A volume of 5 µL and 10 µL of marker were loaded on the left and right well respectively. Marker band sizes are 7.1, 20.6, 28.9, 34.8, 49.1, 80, 124, and 209 kDa.

Supplementary References

1. Hanahan D. Studies on transformation of *Escherichia coli* with plasmids. *J Mol Biol* 166, 557-80.
2. Rosenberg AH, Lade BN, Dao-shan C, Lin SW, Dunn JJ, Studier FW. Vectors for selective expression of cloned DNAs by T7 RNA polymerase. *Gene* 56, 125-135 (1987).
3. Carter P, Bedouelle H, Winter G. Improved oligonudeotide site-directed rautagenesis using M13 vectors. *Nucl Acids Res 13,* 4431-4443 (1985).
4. Salema V, Fernández LÁ. High yield purification of nanobodies from the periplasm of *E. coli* as fusions with the maltose binding protein. *Prot Expr Purif* 91, 42-48 (2013).
5. Piñero-Lambea C, Bodelón G, Fernández-Periáñez R, Cuesta AM, Álvarez-Vallina L, Fernández L. Programming controlled adhesion of *E. coli* to target surfaces, cells, and tumors with synthetic adhesins. *ACS Synth Biol* 4, 463-473 (2015).
6. Salema V, et al. Selection of single domain antibodies from immune libraries displayed on the surface of *E. coli* cells with two β-domains of opposite topologies. *PLoS One* 8, e75126 (2013).
7. Fernández L.A., Enjuanes, L.., de Lorenzo V. Specific secretion of active single-chain Fv antibodies into the supernatants of *Escherichia coli* cultures by use of the hemolysin system. *Appl Env Microbiol* 66, 5024-5029 (2000).
8. Nyerges Á, et al. A highly precise and portable genome engineering method allows comparison of mutational effects across bacterial species. *Proc Natl Acad Sci USA* 113, 2502-2507 (2016).
9. Fraile S, Muñoz A, de Lorenzo V, Fernández LA. Secretion of proteins with dimerization capacity by the haemolysin type I transport system of *Escherichia coli. Mol Microbiol* 53, 1109-1121 (2004).
