# Supplementary material for: ssDNA recombineering boosts in vivo evolution of nanobodies displayed on bacterial surfaces
Source: Commun Biol. 2021 Oct 7;4:1169. doi: 10.1038/s42003-021-02702-0 (PMC8497518; doi:10.1038/s42003-021-02702-0)
Supplement: Supplementary file 3 — Description of Additional Supplementary Files [file 42003_2021_2702_MOESM3_ESM.pdf]

Description of Additional Supplementary Files

File name: Supplementary Data 1

Description: Source data for all the graphs and charts of main figures.
